# Efficacy and safety of Lian-Hua Qing-Wen granule for COVID-2019: A protocol for systematic review and meta-analysis

**DOI:** 10.1097/MD.0000000000020203

**Published:** 2020-06-05

**Authors:** Zhipeng Hu, Maoyi Yang, Chunguang Xie

**Affiliations:** Hospital of Chengdu University of Traditional Chinese Medicine, Sichuan Province, PR China.

**Keywords:** COVID-19, Lian-Hua Qing-Wen granule, protocol, systematic review and meta-analysis

## Abstract

**Background:**

Coronavirus disease 2019 (COVID-19) is a global pandemic caused by the Severe Acute Respiratory Syndrome Coronavirus-2 (SARS-CoV-2). Since the outbreak, the disease has caused more than 60,502 deaths worldwide. Lian-Hua Qing-Wen Granule (LHQWG) is widely used in treating COVID-19 in China. However, there is no evidence that LHQWG is effective for COVID-19.

**Methods and analysis:**

A comprehensive literature search will be conducted. Two methodological trained researchers will read the title, abstract and full texts and independently select the qualified literature according to inclusion and exclusion criteria. After assessment of the risk of bias and data extraction, we will conduct meta-analyses for outcomes related to COVID-19. The heterogeneity of data will be investigated by Cochrane X^2^ and *I*^*2*^ tests. Publication bias assessment will be conducted by funnel plot analysis and Egger test.

**Results:**

The results of our research will be published in a peer-reviewed journal.

**Conclusion:**

Our study aims to systematically present the clinical evidence of LHQWG in treating COVID-19, which will be of significant meaning for further research and clinical practice.

**OSF registration number:**

10.17605/OSF.IO/27SBU.

## 1 Introduction

Coronavirus disease 2019 (COVID-19) is a global pandemic caused by the Severe Acute Respiratory Syndrome Coronavirus-2 (SARS-CoV-2).^[1,2]^ The patients of COVID-19 usually present with fever, cough, while about 23.7% of patients are accompanied by at least one coexisting disease.^[3–7]^ The rapid rise in the number of patients has placed a heavy burden on the health care system.^[8]^ Up to April 6, 2020, the disease has infected 836,769 people and caused 60,502 deaths all over the world.

At present, there is no effective treatment for this disease.^[9]^ The clinical management of this disease mainly depends on supportive treatment. Studies found that some treatments may be effective, but the efficacy remains to be further evaluated.^[10–12]^ Thus, effective treatment is still urgently needed.

In China, traditional Chinese medicine (TCM) is widely used in treating COVID-19.^[13,14]^ It is reported that more than 85% of COVID-19 patients are treated with TCM.^[15]^ Among the many commonly used TCMs, the most commonly used one is Lian-Hua Qing-Wen Granule (LHQWG). LHQWG was first used in the treatment of influenza, and studies have found that the drug is superior to oseltamivir in improving the symptoms of influenza A virus infection.^[16]^ Since the outbreak of COVID-19, this medicine has been widely recommended for clinical treatment of patients with common symptoms. In the fifth edition of COVID-19's diagnosis and treatment guideline issued by the China Health Commission, LHQWG is listed as one of the recommended medicine candidates. Researchers carried out a series of studies on the efficacy of this medicine in the treatment of COVID-19. It was found that routine treatment combined with LHQWG could significantly improve the clinical symptoms including fever, fatigue, cough, sputum, shortness of breath, chest tightness, loss of appetite and so on; suggesting that LHQWG is an effective treatment for COVID-19 patients.^[17–20]^ In addition, the experimental research on the mechanisms of LHQWG in the treatment of COVID-19 by means of cell experiment and network pharmacology is also being widely carried out.^[21–24]^

## 2 Methods and analysis

### 2.1 Study registration

This study has been registered in advance on the website of Open Science Framework (OSF, https://osf.io/) with a registration number of DOI: 10.17605/OSF.IO/27SBU. This systematic review protocol is reported in accordance with Cochrane reporting expectations, as recommended by the Cochrane handbook.^[25]^

### 2.2 Inclusion and exclusion criteria

#### 2.2.1 Study design

In this study, both randomized studies and non-randomized studies will be included. Randomized studies can provide reliable clinical evidence, but it can be time-consuming and expensive. Non-randomize studies may lead to greater bias, but it is more convenient to obtain clinical data. Since COVID19 is an urgent public health event, it is difficult to carry out randomized studies, it is appropriate to include non-randomized studies in this systematic review and meta-analysis.

#### 2.2.2 Participants

Participants with laboratory-confirmed COVID-19 will be included in this study. The assay was primarily RC-qPCR, and there were no restrictions on the age, sex or disease severity of the participants.

#### 2.2.3 Intervention

Studies using LHQWG or Lian-Hua Qing-Wen Capsule will be included. There will be no restriction about the doses and methods of use of intervention. Also, there will be no limitation about control group.

#### 2.2.4 Outcomes

Total clinical effective rate, effective rate of clinical symptoms, disappearance rate of clinical symptoms, treatment time, improvement rate of lung CT, adverse events.

### 2.3 Study search

Three English database including PubMed, EMBASE, Cochrane Library Central Register of Controlled Trials and four Chinese databases including China National Knowledge Infrastructure (CNKI) database, Wanfang Data Knowledge Service Platform, the VIP information resource integration service platform, China Biology Medicine Disc will be searched from its inception to April 6, 2020. There will be no language limitation. Preprinted website including arXiv (http://arxiv.org/), BioRxiv (https://www.biorxiv.org/), F1000 (https://f1000.com/) and PeerJ Preprints (https://peerj.com/preprints/) will also be searched to find out more unpublished manuscript. Chinese Clinical Trial Registry (ChiCTR) and ClinicalTrials.gov will also be searched to find out ongoing research. The references of included manuscript will be searched.

A search strategy of the combination of controlled vocabulary and text words will be adopted. Boolean operators will be used to concatenate search terms. This work will be conducted by 2 authors (Zhipeng Hu and Maoyi Yang) independently. The search strategy of PubMed is presented in Table 1.

**Table 1:**
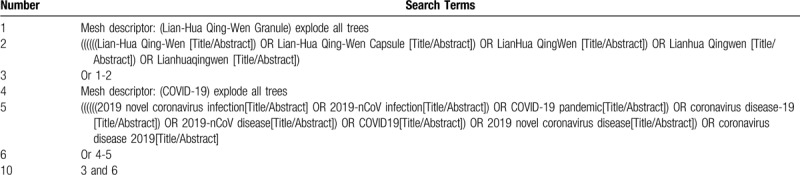
Example of PubMed search strategy.

### 2.4 Study selection

**Figure 1:**
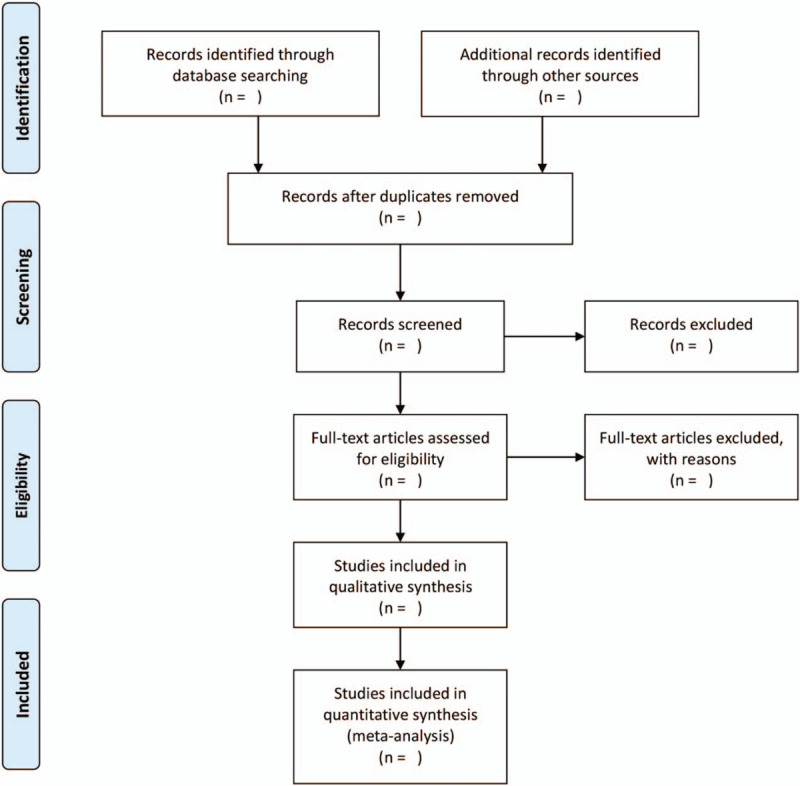
Flow chart of study selection.

EndNote X9 will be used to screen the citations independently according to inclusion and exclusion criteria by two reviewers (Zhipeng Hu and Maoyi Yang). Discrepancies between two authors will be solved by discussion with a third author (Chunguang Xie). A research flow chart will be drawn to show the whole process of research selection (Fig. 1).

### 2.5 Data extraction

Data extraction will be conducted by 2 independent authors (Maoyi Yang and Zhipeng Hu) according to a prespecified form and checked by a third author (Chunguang Xie). The following data will be extracted: the first author's name, publication time, country, article title, article type, interventions in experimental and control group, course of treatment, severity of disease, number of patients in each group, ages and sex of patients, outcomes and adverse effect. If the author does not report certain information in the article, we will then contact the authors by email for more detailed information. Once the extraction is complete, the 2 authors will check with each other to ensure the accuracy of the data.

### 2.6 Risk of bias assessment

Version 2 of the Cochrane risk-of-bias tool for randomized trials (RoB2) and the risk of bias in non-randomized studies of interventions (ROBINS-I) tool will be used to assess the risk of bias of randomized studies and non-randomized studies respectively, as recommended by the Cochrane handbook.^[26,27]^

### 2.7 Data analysis

Data analysis will be conducted using Stata 14.0 software. Risk ratio (RR) or odds ratio (OR) and 95% confidence interval (CI) will be used for dichotomous outcomes and 95% CI and mean difference (MD) or standardized mean difference (SMD) will be used for continuous outcomes. The number needed to treat will be calculated for the interpretation of results. Cochrane *X*^*2*^ and *I*^*2*^ tests will be conducted to assess the heterogeneity analysis between studies. A random effect model will be used if *P* < .05 and *I*^*2*^ > 50%. When *P* > .05 and *I*^*2*^ < 50%, then a fixed effect model will be used to. The results of randomized studies and non-randomized studies will be analyzed and presented independently. Subgroup analysis will be conducted to explore the subgroup effects and investigate the source of heterogeneity. If there is a substantial heterogeneity and quantitative synthesis is not appropriate, the results will be presented in the form of tables and figures.

Non-reporting bias will be evaluated by funnel plot and Egger test.^[28]^ A *P* value less than .05 indicates the existence of publication bias.

## 3 Discussion

The aim of this study was to summarize the efficacy of LHQWG on COVID-19 to provide an accurate guide for further research and clinical application. This study has some highlights. First, in order to collect clinical evidence as comprehensively as possible, preprinted websites will be searched in addition to main databases for systematic review. In addition, given the speed at which the epidemic is developing and the difficulty of conducting clinical trials, we will include both randomized studies and non-randomized studies in the research. Non-randomized studies have more biases and confounding than randomized studies, so the results of the 2 types of studies will be presented separately. In conducting the risk of bias assessment, we will use the latest version of the tool recommended in the handbook, which will methodologically ensure the correctness of our study.

## Author contributions

The protocol was designed by ZH and MY under the guidance of CX. All the authors participated in the study. The manuscript was drafted by ZH and revised by CX. All authors approved the final manuscript before submission. ZH and MY contributed equally to this work and should be regarded as co-first authors.

**Conceptualization**: Zhipeng Hu, Chunguang Xie.

**Data curation**: Zhipeng Hu, Maoyi Yang.

**Formal analysis**: Zhipeng Hu, Maoyi Yang.

**Investigation**: Zhipeng Hu, Maoyi Yang.

**Methodology**: Zhipeng Hu, Maoyi Yang.

**Project administration**: Chunguang Xie

**Software**: Zhipeng Hu, Maoyi Yang.

**Visualization**: Zhipeng Hu, Maoyi Yang.

**Writing – original draft**: Zhipeng Hu.

**Writing – review and editing**: Chunguang Xie.
